# Two novel UPLC methods utilizing two different analytical columns and different detection approaches for the simultaneous analysis of velpatasvir and sofosbuvir: application to their co-formulated tablet

**DOI:** 10.1186/s13065-019-0635-2

**Published:** 2019-09-30

**Authors:** Moustapha Eid Moustapha, Rania Mohamed El-Gamal, Fathalla Fathalla Belal

**Affiliations:** 1 Department of Chemistry, College of Science and Humanities, Prince Sattam Bin-Abdul Aziz University, Al-Kharj, 11942 Kingdom of Saudi Arabia; 2 Department of Pharmaceutical Analytical Chemistry, Faculty of Pharmacy, Mansoura University, P.O. Box 35516, Mansoura, Egypt; 3 Present Address: College of Pharmacy, Prince Sattam Bin-Abdul Aziz University, King Abdullah Road, Al-Kharj, Kingdom of Saudi Arabia

**Keywords:** Velpatasvir, Sofosbuvir, UPLC, Fluorescent detection, UV-spectrophotometric detection

## Abstract

In the present study two different RSLC columns, Acclaim RSLC 120 C18, 5.0 µm, 4.6 × 150 mm (column A) and Acclaim RSLC 120 C18, 2.2 µm, 2.1 × 100 mm (Column B) were utilized for the analysis of velpatasvir (VPS) in presence of sofosbuvir (SFV), where due to the encountered fluorescent properties of VPS fluorescent detection at 405 nm after excitation at 340 nm (Method 1) was used for its detection where the non-fluorescent SFV did not interfere. The same columns were further utilized for the simultaneous determination of SFV and VPS either in bulk form or in their combined tablet, where UV- spectrophotometric detection at 260 nm was selected for the simultaneous analysis of both drugs (Method 2). A mobile phase consisting of NaH_2_PO_4_, pH 2.5 (with phosphoric acid) and acetonitrile in a ratio of 60:40 v/v was used for both methods. The mobile phase was pumped at a flow rate of 1.0 mL/min when using column, A and 0.5 mL/min when using column B. The methods showed good linearity over the concentration ranges of 1.0–5.0 and 2.5–10.0 ng/mL for VPS when utilizing Method 1 A and B respectively. Where the linearity concentration range was from 30.0–150.0 to 120–600.0 ng/mL for VPS and SFV respectively when applying Method 2. Both methods 1 and 2 were performed by utilizing the two analytical columns. The different chromatographic parameters as retention time, resolution, number of theoretical plates (N), capacity factor, tailing factor and selectivity were carefully optimized. The results show that comparing the performance of the two utilized columns revealed that shorter column (2.1 mm × 100 mm) with small particle packing was superior to the longer column (4.6 × 150 mm) for the analysis of the studied drugs allowing a reduction of the analysis time by 70% without any detrimental effect on performance. This prompts the decrease of the investigation costs by saving money on organic solvents and expanding the overall number of analyses per day.

## Introduction

**Fig. 1:**
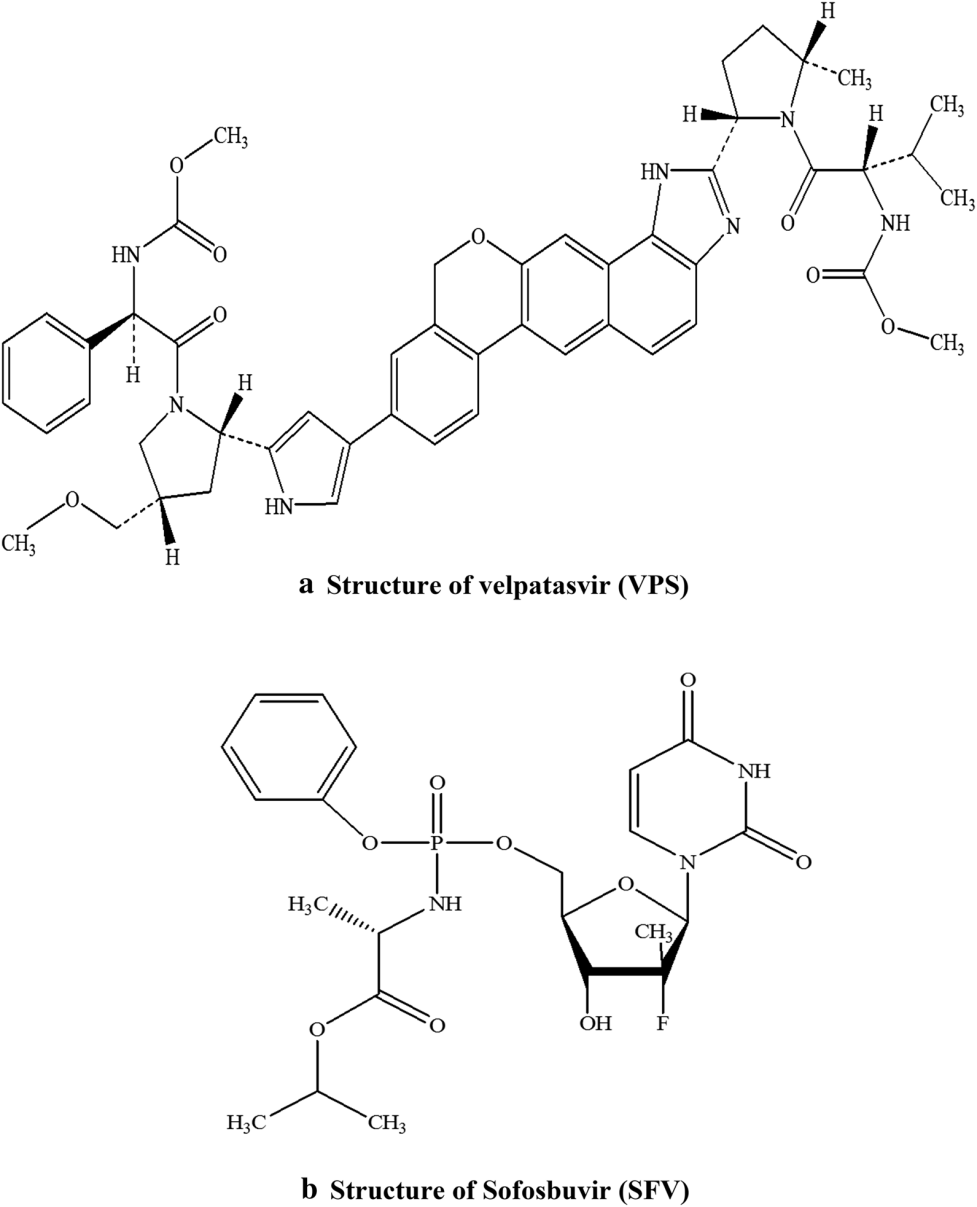
The structural formulae of the studied drugs. **a** Velpatasvir (VPS), **b** sofosbuvir (SFV)

Hepatitis C is an infectious liver disease caused by infection with Hepatitis C Virus (HCV) that is considered a very dangerous disease, influencing about from three to five million people in the United States (US) and about one hundred and seventy million people worldwide. This disease is asymptomatic in its early stages however if it becomes chronic it might prompt risky perilous inconveniences, including liver failure, hepatocellular carcinoma and mortality [1]. Velpatasvir (VPS) is methyl {(2S)-1-[(2S,5S)-2-(9-{2-[(2S,4S)-1-{(2R)-[(methoxycarbonyl)amino]-2-phenylacetyl}-4(methoxymethyl)pyrrolidin-2-yl]-1H-imidazol-4-yl}-1,11 dihydro [2] benzopyrano[4′,3′:6,7]naphtho[1,2-d]imidazol-2-yl)-5-methylpyrrolidin-1-yl]-3-methyl-1-oxobutan-2-yl}carbamate, Fig. 1a.

VPS is a Direct-Acting Antiviral (DAA) medication that plays a significant role in the combination therapy of chronic Hepatitis C. HCV is a solitary stranded RNA virus with nine particular genotypes, where, genotype 1 is the most widely recognized type in the United States, and influencing more than 70% of patients suffering from chronic HCV. Since 2011, the presentation of Direct Acting Antivirals (DAAs, for example, VPS) have fundamentally improved chronic hepatitis C treatment. One of the major advantage of VPS is that it has a noteworthy raised boundary to resistance than its previous generation of NS5A inhibitors, as daclatasvir and ledipasvir, this accounts for its high potency and efficacy as a treatment for chronic Hepatitis C [2]. Sofosbuvir (SFV) (isopropyl (2S)-2-[(2R,3R,4R,5R)-5-(2, dioxopyrimidin-1-yl)-4-fluoro-3-hydroxy-4-methyl-tetra hydrofuran-2-yl] methoxy-phenoxy-phosphoryl] amino] pro-panoate) is a nucleotide analog NS5B polymerase inhibitor. SFV is a prodrug that is mainly used for the treatment of HCV, either alone or in combination with other drugs like, VPS, ribavirin, and ledipasvir [3] (Fig. 1b).

In June 2016, the American Association for the Study of Liver Diseases (AASLD) and the Infectious Diseases Society of America (IDSA) approved VPS and SFV combination (Epclusa) as 1st line therapy for the different six genotypes of Hepatitis C [4].

Since the drugs are recently approved, their literature revealed few analytical methods reported up to date, where, SFV alone was determined by applying chromatographic and spectrophotometric techniques [5, 6]. The forced degradation behavior of SFV was investigated by mean of liquid chromatography-tandem mass spectrometry (LC–MS/MS) [7]. Few UPLC-MS/MS techniques were utilized for the simultaneous analysis of SFV and other antiviral drugs like ribavirin, ledipasvir or in presence of its metabolite [8–10].

Different RP-HPLC methods were reported for the simultaneous determination of SFV and VPS either in bulk, combined tablets or biological fluids [11–14], in addition to two spectrofluorometric methods that were recently reported for the assay of VPS in pharmaceutical tablets and body fluids [15, 16].

The main objective of this work was to develop novel UPLC methods for the simultaneous analysis of VPS and SFV utilizing different analytical columns and different detection approaches.

## Experimental

### Apparatus

Chromatographic analyses were performed using Thermo Scientific DIONEX UltiMate 3000 UHPLC Rapid Separation System (Thermo Fisher Scientific Inc., MA, USA), connected to a quaternary rapid separation pump (LPG-3000RS), Ultimate 3000RS autosampler (WPS-3000), rapid separation diode array detector (DAD-3000RS) and rapid separation fluorescence detector (DIONEX Ultimate 3000 RS Flourescence). Data acquisition, peak integration and calibrations were carried out using UHPLC, CHROMELEON7 software, Dionex, Thermo Fisher Scientific, USA. Mobile phases were filtered using Whatman^®^ Nylon membrane filters 0.2 µm, ø47 mm. The mobile phase was degassed with a sonicator of type GT SONIC QTD-series units with digital timer and heater features, GuangDong GT Ultrasonic Co., Ltd, China. Separation was carried on an Acclaim RSLC 120 C18 2.2um 120A (2.1 × 100 mm) and Acclaim RSLC 120 C1 5.0um 120A (4.6 × 150 mm) (Dionex, USA). Ultrapure water was obtained from an Evoqua Ultra Clear TP TWF EDI UV UF TM system, Evoqua Water Technologies, USA.

### Materials and reagents

All solvents used in this work were of HPLC grade. Ultrapure water was used for all preparations. VPS (≥ 98%) was purchased from BioVision, Milpitas Boulevard, Milpitas, CA 95035 USA). SFV (99.98 ± 0.741) was obtained from Cayman chemical company, Ann Arbor, USA) [8]. Acetonitrile and methanol (HPLC grade) were obtained from Merck (Germany). Phosphoric acid, analytical grade Merck (Germany). Sodium dihydrogen phosphate (NaH_2_PO_4_) was obtained from central drug house (CDH), New Delhi, India. Phosphoric acid (0.2 mol/L) solution was used to adjust pH to 2.5.

### Dosage form

Epclusa^®^ (sofosbuvir 400 mg/velpatasvir 100 mg) tablets was manufactured by Gilead Sciences International, Cambridge, UK.

### Standard solutions

Stock solutions of concentration 100.0 μg/mL of VPS and SFV were prepared by dissolving 10 mg of pure drug in 100 mL methanol using an ultrasonic bath. Working standard solutions were prepared by suitable dilution of the stock solutions with mobile phase. All solutions were stored in the refrigerator to keep their stability.

### Chromatographic conditions

Acclaim RSLC columns 120 C18 (120A 4.6 × 150 mm, 5.0um) and Acclaim RSLC 120 C18 (120A 2.1 × 100 mm, 2.2um) were used for methods A and B respectively. A mobile phase consisting of NaH_2_PO_4_, pH 2.5 (with phosphoric acid, 0.2 M) and acetonitrile in a ratio of 60:40 v/v was used for both methods. The mobile phase was vacuum-membrane filtered through a 0.45 μm Millipore membrane filter and degassed for approximately 10 min before use. The flow rate was 1.0 mL/min when using column, A and 0.5 mL/min when using column B. Columns temperature was maintained at 25 °C. For fluorescence detection of VPS, the detector was set at 340/405 nm (Method 1 A and 1B). While for UV detection of both VPS and SFV the detector was set at 260 nm (Method 2A and 2B). The injection volume was 10 uL.

#### Laboratory prepared mixture analysis

Stock solution of (SFV andVPS) was prepared at the ratio of (4:1), where, 40 and 10 mg of both SFV and VPS were quantitatively transferred to 100 mL volumetric flask and the volume was adjusted with methanol. Working standard solutions were prepared by suitable dilution of the stock solution with mobile phase.

Analysis of the working standard solution was accomplished via adapting procedures cited under “Calibration graph construction” section, where, corresponding drug concentrations were calculated from the derived regression equations.

#### Calibration graph construction

A calibration curve was created by accurately measuring volumes of the appropriate drugs working standard solutions delivered into a series of 10 mL volumetric flasks in order to prepare a set of standard solutions in the range specified by the method. The standard solutions were completed to volume with the mobile phase and mixed thoroughly. Aliquots of 10 μL were injected (triplicate) into the columns and eluted with the mobile phase under the optimum chromatographic conditions. The peak area was plotted against the concentration of the drug in ng/mL. Consequently, the corresponding regression equations were derived.

#### Procedures for tablets

A precise weight of the blended content of 10 powdered tablets equal to 10.0 mg of VPS and 40.0 mg of SFV was quantitatively conveyed into a 100 mL volumetric flask and around 30 mL methanol was added. The flask contents were sonicated for 30 min, and made to 100 mL with the same solvent. The solution was filtered through cellulose acetate syringe filter. Working standard solutions were prepared by suitable dilution of the filtered solution with mobile phase.

Analysis of the working standard solution was accomplished via adapting procedures cited under “Calibration graph construction” section, where, the nominal contents of the tablet were calculated from the derived regression equations or the calibration curve.

## Results and discussion

**Fig. 2:**
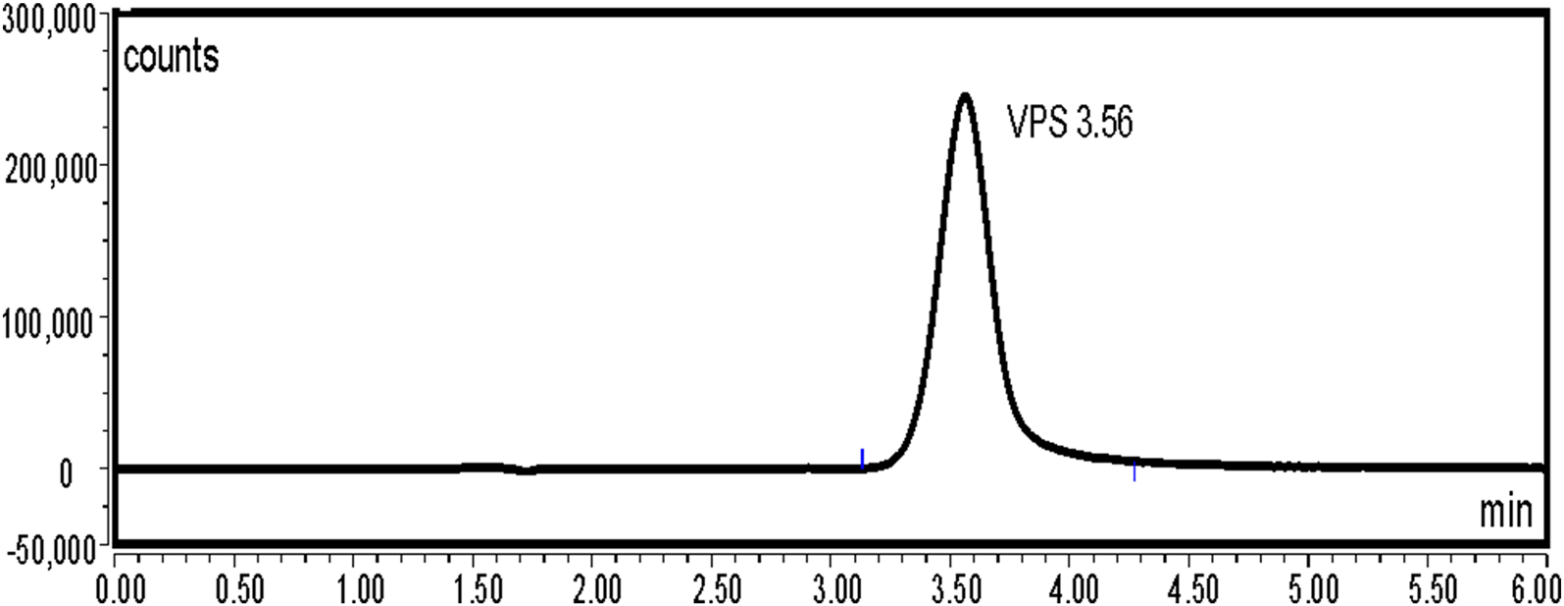
Typical chromatograms of VPS 2.5 ng/mL under the described chromatographic conditions (Method 1 A)

**Fig. 3:**
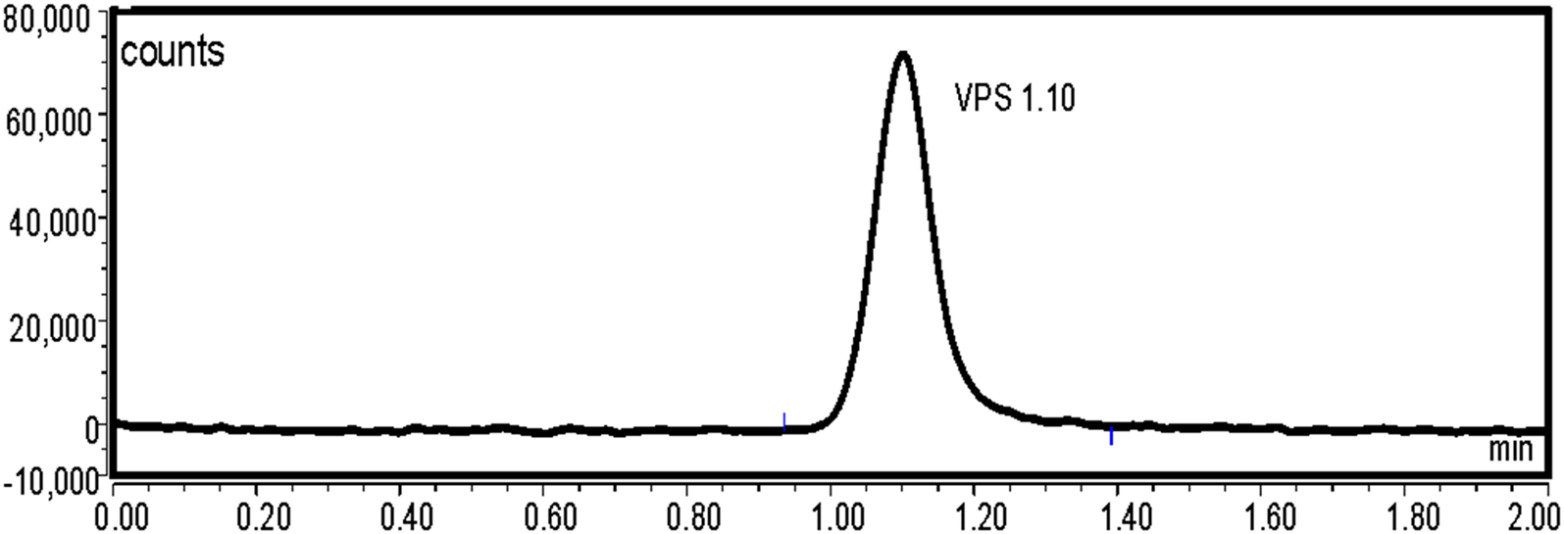
Typical chromatograms of VPS 1.0 ng/mL under the described chromatographic conditions (Method 1 B)

**Fig. 4:**
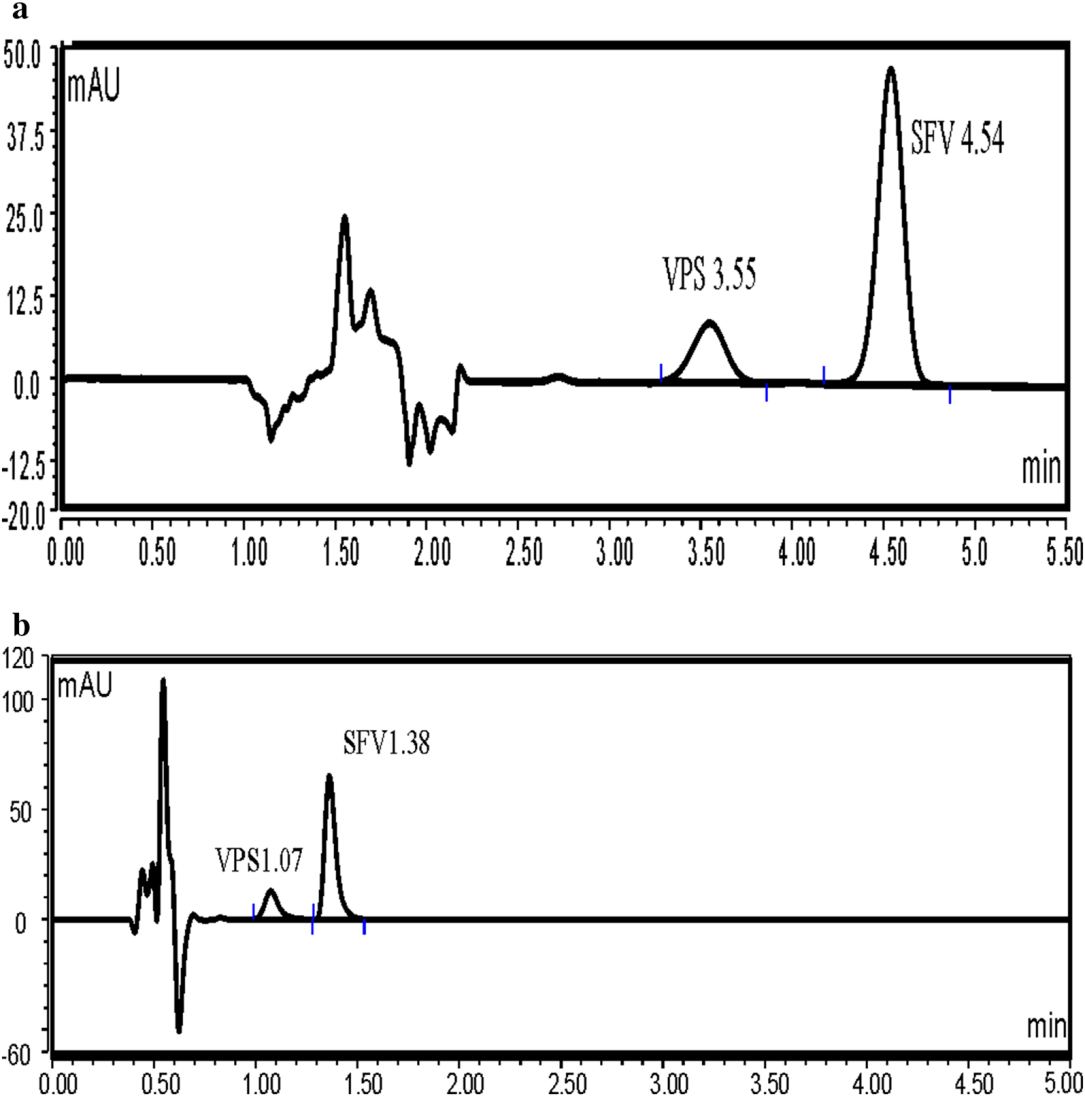
Typical chromatogram of a laboratory prepared mixture of VPS (10 ng/mL) and SFV (40 ng/mL) under the described chromatographic conditions (Method 2 A and B). **a** Studied drugs in the mobile phase utilizing column A. **b** Studied drugs in the mobile phase utilizing column B

VPS was found to exhibit an intense fluorescence at 405 nm, after excitation at 340 nm. As a consequence, we aimed to utilize this emission band using UPLC coupled with fluorescence detection, to develop a new method for its analysis in presence of SFV, the method was applied for the analysis of the VPS (pure form) in presence of SFV (Method 1) (Figs. 2 and 3). Moreover, an UPLC with UV detection was utilized for the simultaneous analysis of VPS and SFV in their pure form as well as in their combined tablet (Method 2) (Fig. 4a, b). Both methods 1 and 2 were performed utilizing two different analytical columns.

### Optimization of experimental conditions

#### Choice of appropriate wavelength

VPS was reported to exhibit a very strong fluorescence permitting very sensitive detection. The optimum excitation and emission wavelengths were determined via preliminary scanning of its fluorescence in the mobile phase, VPS was found to exhibit maximum fluorescence intensity at 405 nm after excitation at 340 nm (Fig. 2).

For simultaneous analysis of VPS and SFV, their λ_max_ were determined through spectrophotometric scan where 260 nm was chosen as optimum wavelength for their simultaneous determination.

#### Mobile phase composition

Several modifications in the mobile phase composition were carried out in a trial to optimize the selectivity, efficiency, and resolution of the chromatographic system. These modifications involved, the pH of the mobile phase, the type and ratio of the organic modifier, column temperature and the flow rate. The results achieved are summarized in Tables 1 and 2.

**Table 1:** Optimization of the chromatographic conditions for determination of VPS by Method 1

Parameter	No. of theoretical plates (N)		Capacity factor (k′)		Tailing factor (T_f_)	
	Column (A)	Column (B)	Column (A)	Column (B)	Column (A)	Column (B)
Column temperature °C						
Room temperature	2900.3	406.2	1.839	2.675	0.532	0.831
30 °C	2941.6	–	1.987	–	0.660	–
40 °C	4547.4	1009.7	2.183	2.038	0.536	0.900
50 °C	5030.4	644.4	2.348	3.179	0.517	0.877
60 °C	–	799.3	–	3.454	–	0.908
pH of mobile phase						
2.5	2900.3	406.2	1.839	2.675	0.532	0.831
3.3	3921.4	408.2	2.744	4.150	0.528	0.947
4.0	3305.7	410.6	3.976	6.425	0.491	0.924
Type of organic modifier of Conc 40% (v/v)						
Acetonitrile	2720.4	378.8	1.831	2.583	0.556	0.867
Methanol	1140.1	365.4	1.843	2.621	0.514	0.907
Ethanol	2304.3	352.9	1.870	2.592	0.413	0.871
Ratio organic modifier: mobile phase (acetonitrile) (v/v)						
40:60	3145.5	372.7	1.824	2.554	0.525	0.825
60:40	2720.4	378.8	1.831	2.583	0.556	0.867
80:20	2386.9	314.5	1.844	2.642	0.552	0.906
90:10	2210.2	316.7	1.848	2.654	0.575	0.866
Effect of flow rate (mL/min)						
0.3	–	452.8	–	4.613	–	0.768
0.5		205.0		1.433		0.750
1.0	3551.6		2.376		0.571	
1.2	2900.3	–	1.839	–	0.532	–

$$ {\text{Number of theoretical plates }}\left( {\text{N}} \right)\, = \,5.54\left( {\frac{{{t}_{{R}} }}{{{Wh}/2}} } \right)^{2} $$

$$ {\text{Tailing factor }}\left( {\text{T}} \right)\, = \,\frac{{{W}_{0.5} }}{{2{f}}} {\text{k}^{\prime}} \, = \,{{({\text{tR}} {-}{\text{ t}}0)} \mathord{\left/ {\vphantom {{({\text{tR}} {-}{\text{ t}}0)} {{\text{t}}0}}} \right. \kern-0pt} {{\text{t}}0}} $$,

**Table 2:** Optimization of the chromatographic conditions for the determination of SFV by Method 2

Parameter	No. of theoretical plates (N)		Capacity factor (k′)		Tailing factor (T_f_)	
	Column (A)	Column (B)	Column (A)	Column (B)	Column (A)	Column (B)
Column temperature °C						
25 °C	4354.9	1014.2	1.147	4.638	0.475	0.629
40 °C	2950.3	756.4	1.057	4.404	0.506	0.772
50 °C	2620.8	677.9	0.989	4.208	0.471	0.73
65 °C	1895.1	468.9	0.871	3.792	0.468	0.679
pH of mobile phase						
2.5	4354.9	1014.2	1.147	4.638	0.475	0.629
3.5	3981.2	1089	1.145	4.667	0.526	0.588
5	4617.2	1066.9	1.145	4.667	0.455	0.572
Type of organic modifier of Conc 40% (v/v)						
Acetonitrile	4390.4	1004.5	1.236	4.667	0.446	0.679
Methanol	5073.4	1214.5	1.179	4.738	0.474	0.664
Ethanol	3342	925.4	1.175	4.708	0.481	0.65
Ratio organic modifier: mobile phase (Acetonitrile) (v/v)						
40:60	4354.9	1100.3	1.147	4.696	0.475	0.677
60:40	1925.3	1004.5	1.173	4.667	0.453	0.679
80:20	7501.7	1648.5	1.187	4.75	0.468	0.647
90:10	8304.6	2064.8	1.19	4.792	0.458	0.653
Effect of flow rate (mL/min)						
0.3		1041		8.196		0.816
0.5		1090.7		4.613		0.613
1	5093.6		1.53		0.287	
1.2	1117.9		1.175		0.465	

$$ {\text{Number of theoretical plates }}\left( {\text{N}} \right)\, = \,5.54\left( {\frac{{{t}_{{R}} }}{{{Wh}/2}} } \right)^{2} $$

$$ {\text{Tailing factor }}\left( {\text{T}} \right)\, = \,\frac{{{W}_{0.5} }}{{2{f}}}{\text{k}^{\prime}} \, = \,{{({\text{tR}} {-}{\text{ t}}0)} \mathord{\left/ {\vphantom {{({\text{tR}} {-}{\text{ t}}0)} {{\text{t}}0}}} \right. \kern-0pt} {{\text{t}}0}} $$,

##### pH of the mobile phase

The influence of the pH change on the different chromatographic parameters studied was investigated via changing the pH of the mobile phase and monitoring the consequence change in parameter.

For both methods pH of 2.5 was the optimum pH resulting in a well-defined peak, optimum resolution of both drugs by Method 2 and shortest analysis time.

##### Type of organic modifier of Conc 40% (v/v)

Different organic modifiers of concentration 40% (v/v) were utilized in this study. These include acetonitrile, methanol and ethanol. It was found that acetonitrile was the organic modifier of choice for both methods resulting in highest number of theoretical plates, maximum resolution and least tailing factor.

##### Concentration of organic modifier

To study the influence of the concentration of acetonitrile on the proposed analysis methods, its concentration was varied over the range of (40–90%, v/v). As the percentage of acetonitirle increases in the mobile phase a marked peak broadening was noticed, with a concomitant decrease in the number of theoretical plates. Hence, a concentration of 40% acetonitrile was selected as the optimal concentration where it provides an optimum combination of peak symmetry, resolution factor and analysis time (Tables 1, 2).

##### Flow rate

The influence of flow rate on the retention time and peak shape was investigated for both methods with utilization of the two comparative columns.

A flow rate of 1.0 mL/min was optimal for both methods when using column, A, where a flow rate of 0.5 mL/min was optimal for good separation within a reasonable elution time when using column B. This is mainly due to the increased back pressure observed when pumping a mobile phase through columns with small particle size packing.

##### The effect of column temperature

The column temperature was altered through the study to attain the suitable temperature for maximum resolution and optimal peak symmetry. Column temperature was varied over the range (30–60 °C), it was found that room temperature was optimal resulting in highest number of theoretical plates, minimal tailing and best resolution (Tables 1, 2).

### Method validation

The validity of the proposed UPLC methods was examined in terms of linearity, ranges, limits of detection, limits of quantification, accuracy, precision, robustness, specificity, stability of standard solutions and mobile phase.

#### Linearity and range

Under the above-demonstrated experimental conditions, a linear relationship was obtained by plotting the peak areas against the drugs concentrations. The graphs were found to be rectilinear over the concentration ranges referred to in Tables 3, 4.

**Table 3:** Analytical performance data for the determination of VPS by Method 1

Parameter	Value	
	Column (A)	Column (B)
Linearity and range (ng/mL)	1.0–5.0	2.5–10.0
Correlation coefficient (r)	1.0	0.9999
Slope	20,723.82	49,677.50
Intercept	− 745.284	− 21,790.770
S_y/x_, SD of the residuals	346.597	1808.52
S_a_, SD of the intercept	363.51	1871.66
S_b_, SD of the slope	109.60	296.12
SD	0.89	0.97
%RSD^a^	0.888	0.97
%Error^b^	0.398	0.435
LOD^c^	0.06	0.12
LOQ^d^	0.18	0.38

^a^Percentage relative standard deviation

^b^Percentage relative error

^c^Limit of detection

^d^Limit of quantitation

**Table 4:** Analytical performance data for the determination of SFV by Method 2

Parameter	SFV		VPS	
	Column (A)	Column (B)	Column (A)	Column (B)
Linearity and range (ng/mL)	120–600	120–600	30–150	30–150
Correlation coefficient (r)	1.0000	1.0000	0.9998	0.9999
Slope	0.029	0.029	0.013	0.038
Intercept	− 0.009	− 0.016	− 0.003	− 0.063
S_y/x_ SD of the residuals	0.036	0.023	0.014	0.011
S_a_ SD of the intercept	0.034	0.022	0.014	0.010
S_b_ SD of the slope	0.0001	0.0001	0.0002	0.0001
SD	0.48	0.28	2.16	0.25
%RSD^a^	0.483	0.283	2.156	0.252
%Error^b^	0.216	0.127	0.965	0.113
LOD^c^	3.94	2.47	3.41	0.90
LOQ^d^	11.94	7.47	10.33	2.74

^a^Percentage relative standard deviation

^b^Percentage relative error

^c^Limit of detection

^d^Limit of quantitation

Statistical analysis [17] of the data showed high values of the correlation coefficient (r) of the regression equation, minute values of the standard deviation of residuals (Sy/x), of intercept (Sa) and of slope (Sb), and small value of the percentage relative standard deviation and the percentage relative error (Tables 3, 4). These values demonstrated the linearity of the alignment diagrams.

#### Limits of quantitation and limits of detection

Limits of quantitation (LOQ) and limits of detection (LOD) were evaluated according to ICH Q2R1 recommendations using the following equation [18]:

$$ {\text{LOQ}} = {\text{10Sa/b and LOD}}  = 3.3 {\text{Sa/b}} $$

where Sa = standard deviation of the intercept of the calibration curves and b = slope of the calibration curves. The values of LOD and LOQ are summarized in Tables 3 and 4.

#### Accuracy

To demonstrate the accuracy of the proposed techniques, the results of the assay of the studied drugs were contrasted with those of the comparison HPLC method [11]. Statistical analysis of the results using Student’s *t* test and variance ratio F-test [17] uncovered no huge distinction between the performance of the methods in regard to accuracy and precision, individually (Tables 5, 6).

**Table 5:** Application of Method 1 for the analysis of VPS in its pure forms

Studied drug	Proposed method						Comparison method [11]
	Amount taken (ng/mL)		Amount found (ng/mL)		% Found		% Found
	Column (A)	Column (B)	Column (A)	Column (B)	Column (A)	Column (B)	
VPS	1.0	2.5	1.016	2.469	101.64	98.79	101.12
	2.0	3.5	1.989	3.551	99.47	101.47	102.45
	3.0	5.0	2.987	4.980	99.57	99.60	99.78
	4.0	7.5	3.992	7.497	99.80	99.96	101.77
	5.0	10.0	5.015	10.002	100.30	100.02	99.14
Mean					100.16	99.97	100.85
± SD					0.89	0.97	1.37
t-test					0.95	1.17	(2.31)*
F-test					2.39	1.99	(6.39)*

Each result is the average of three separate determinations

* Figures between parentheses are the tabulated t and F values at P = 0.05 [17]

**Table 6:** Application of Method 2 for the determination of the studied drugs in their pure form

Studied drug	Proposed method						Comparison method [11]
	Amount taken (ng/mL)		Amount found (ng/mL)		% Found		% Found
	Column (A)	Column (B)	Column (A)	Column (B)	Column (A)	Column (B)	
SFV	120.0	120.0	121.208	120.765	101.01	100.64	100.61
	200.0	200.0	200.205	199.979	100.10	99.99	99.71
	280.0	280.0	279.372	279.766	99.78	99.92	99.82
	400.0	400.0	399.597	400.431	99.90	100.11	99.89
	600.0	600.0	601.597	600.989	100.27	100.16	100.06
Mean					100.21	100.16	100.02
± SD					0.48	0.28	0.354
t-test					0.72	0.72	(2.31)*
F-test					1.86	1.57	(6.39)*
VPS	30.0	30.0	29.134	30.045	97.11	100.15	96.41
	50.0	50.0	51.597	49.907	103.19	99.81	98.24
	70.0	70.0	69.821	70.056	99.74	100.08	98.10
	100.0	100.0	100.119	99.523	100.12	99.52	99.58
	150.0	150.0	150.321	149.981	100.21	99.99	99.47
Mean					100.07	99.91	98.36
± SD					2.16	0.25	1.29
t-test					2.06	0.95	(2.78)*
F-test					2.82 (6.39)*	0.0009 (0.156)*	

Each result is the average of three separate determinations

* Figures between parentheses are the tabulated t and F values at P = 0.05 [17]

#### Precision

The intraday precision was assessed through repeat investigation of various concentrations of the studied drugs in pure form within the explicit working concentration ranges.

Each sample was investigated three consecutive times. Likewise, the interday precision was assessed through triplicate examination of the three specified concentrations on three progressive days. The results for both intraday and interday are summarized in Tables 5 and 6. The relative standard deviations were found to be very deliberate showing sensible repeatability and intermediate precision of the proposed techniques (Tables 7, 8, 9).

**Table 7:** Precision data for the determination of VPS by Method 1

Parameters	Column (A)			Column (B)		
	Concentration (ng/mL)			Concentration (ng/mL)		
	1.0	3.0	5.0	2.5	5.0	10.0
Intraday						
% Found	96.83	98.30	99.75	94.23	93.28	99.21
	97.78	98.77	99.71	92.00	91.55	98.72
	94.82	97.66	99.52	93.23	96.26	98.60
($\overline{{\text{x}}}$)	96.48	98.24	99.66	93.15	93.70	98.84
± SD	1.51	0.56	0.12	1.12	2.38	0.32
%RSD	1.5.7	0.57	0.12	1.2	2.54	0.33
%Error	0.9	0.33	0.07	0.69	1.47	0.19
Inter-day						
% Found	98.24	98.30	99.72	92.97	96.61	99.30
	99.44	96.31	100.42	100.22	99.13	100.66
	99.44	96.31	100.42	93.72	99.51	99.79
($\overline{{\text{x}}}$)	99.04	96.97	100.19	95.46	98.42	99.92
± SD	0.69	1.15	0.40	3.99	1.58	0.69
%RSD	0.70	1.19	0.40	4.17	1.60	0.69
%Error	0.40	0.68	0.23	2.41	0.92	0.40

Each result is the average of three separate determinations

**Table 8:** Precision data for the determination of VPS by Method 2

Parameters	Column (A)			Column (B)		
	Concentration (ng/mL)			Concentration (ng/mL)		
	30	70	150	30	70	150
Intraday						
% Found	95.70	99.06	99.83	101.05	99.51	99.77
	93.62	97.15	99.76	92.25	96.37	97.52
	100.94	100.58	99.79	95.06	97.59	99.50
($\overline{{\text{x}}}$)	96.75	98.93	99.79	96.12	97.82	98.93
± SD	3.77	1.72	0.04	4.50	1.58	1.23
%RSD	3.90	1.74	0.04	4.68	1.62	1.24
%Error	2.25	1.00	0.02	2.70	0.93	0.72
Inter-day						
% Found	98.69	97.19	99.92	100.19	98.41	100.21
	101.55	98.21	100.13	96.79	103.68	97.25
	97.43	98.37	99.87	97.74	98.49	99.77
($\overline{{\text{x}}}$)	99.22	97.92	99.97	98.24	100.19	99.80
± SD	2.11	0.64	0.14	1.75	3.02	1.60
%RSD	2.13	0.65	0.14	1.79	3.01	1.61
%Error	1.23	0.38	0.08	1.03	1.74	0.93

Each result is the average of three separate determinations

**Table 9:** Precision data for the determination of SFV by Method 2

Parameters	Column (A)			Column (B)		
	Concentration (ng/mL)			Concentration (ng/mL)		
	30	70	150	30	70	150
Intraday						
% Found	98.69	99.41	99.82	99.47	99.77	99.93
	103.37	101.32	100.28	100.54	99.53	100.10
	100.70	99.55	100.05	100.84	99.95	100.08
($\overline{{\text{x}}}$)	100.92	100.09	100.05	100.28	99.75	100.04
± SD	2.35	1.07	0.23	0.72	0.21	0.09
%RSD	2.33	1.06	0.23	0.72	0.21	0.09
%Error	1.34	0.61	0.13	0.42	0.12	0.05
Inter-day						
% Found	100.34	99.79	99.86	103.21	97.25	100.68
	100.35	99.96	100.01	97.48	95.95	99.91
	100.38	99.77	99.98	98.07	98.73	99.75
($\overline{{\text{x}}}$)	100.36	99.84	99.95	99.59	97.31	100.11
± SD	0.02	0.10	0.08	3.15	1.39	0.50
%RSD	0.02	0.10	0.08	3.17	1.43	0.50
%Error	0.01	0.06	0.05	1.83	0.83	0.29

Each result is the average of three separate determinations

#### Robustness

For the assessment of the techniques robustness, one chromatographic parameter was varied while maintaining all others unaltered. The contemplated variables included; concentration of organic modifier (40% ± 0.1) and pH of the mobile phase (2.5 ± 0.1). These minor changes did not affect the chromatographic separation or the resolution of the studied drugs from each other.

#### Specificity

Specificity is the capability to estimate unequivocally the analytes in presence of other components that might be present [18]. Methods specificity was assessed by investigating diverse laboratory prepared mixtures of VPS and SFV at their specified pharmaceutical ratio (Tables 10, 11). It was additionally demonstrated by its capacity to determine VPS and SFV in their pharmaceutical tablets without interference from regular excipients.

**Table 10:** Assay results for the determination of VPS in laboratory prepared mixture with SFV at their pharmaceutical ratio by Method 1

Combination	Proposed method						Comparison method [11]
	Amount taken (ng/mL)		Amount found (ng/mL)		% Found		% Found
	Column (A)	Column (B)	Column (A)	Column (B)	Column (A)	Column (B)	VPS
SFV/VPS mixture 4:1 (w/w)	1.0	2.5	0.989	2.495	98.93	99.78	101.45
	2.0	3.5	1.899	3.412	94.99	97.50	100.47
	3.0	5.0	2.947	4.913	98.22	98.26	99.12
	4.0	7.5	3.933	7.356	98.34	98.09	
	5.0	10.0	5.003	9.996	100.07	99.96	
Mean					98.11	98.72	100.35
± SD					1.89	1.09	1.17
t-test					− 1.82	1.99	(2.45)*
F-test					2.62 (19.24)*	1.15 (6.94)*	

Each result is the average of three separate determinations

* The figures between parentheses are the tabulated t and F values at P = 0.05 [17]

**Table 11:** Assay results for the determination of VPS and SFV in their laboratory prepared mixture at their pharmaceutical ratio by Method 2

Studied drug	Proposed method						Comparison method [11]
	Amount taken (ng/mL)		Amount found (ng/mL)		% Found		% Found
	Column (A)	Column (B)	Column (A)	Column (B)	Column (A)	Column (B)	
SFV	120	120	120.468	119.472	100.39	99.56	99.98
	200	200	200.140	195.560	100.07	97.78	98.93
	280	280	279.720	273.000	99.90	97.50	100.83
	400	400	399.520	393.600	99.88	98.40	
	600	600	588.120	600.420	98.02	100.07	
Mean					99.65	98.66	100.85
±SD					0.93	1.12	0.95
t-test					0.38	1.61	(2.45)*
F-test					1.04	1.38	(6.94)*
VPS	30	30	30.036	29.817	100.12	99.39	101.45
	50	50	49.420	50.305	98.84	100.61	100.47
	70	70	68.768	70.455	98.24	100.65	99.12
	100	100	99.140	100.130	99.14	100.13	
	150	150	149.910	149.100	99.94	99.40	
Mean					99.26	100.04	100.35
±SD					0.78	0.62	1.17
t-test					1.61	0.50	(2.45)*
F-test					2.25	3.56	(6.94)*

Each result is the average of three separate determinations

* The figures between parentheses are the tabulated t and F values at P = 0.05 [17]

#### Stability of standard solutions and mobile phase

Stock solution stability was studied and evaluated by quantitation of the drugs in comparison to freshly prepared standard solutions. No remarkable variation was noticed in the response to standard solutions, compared to freshly prepared standards. Furthermore, the stability of the mobile phase was examined in a similar method. In both methods the results demonstrated that sample solutions and mobile phase applied during the analysis were stable up to 3 days when preserved in the refrigerator at 4 °C.

### Applications

#### Analysis of VPS and SFV in a laboratory prepared mixture of their pharmaceutical ratio

The reported procedures were effective and applicable for the analysis of VPS in a laboratory prepared mixture with SFV in addition to their simultaneous determination at their pharmaceutical ratio (1:4), as well. The experimental results obtained are expressed in Tables 12 and 13. The concentrations of each compound in the synthetic mixture were evaluated according to the linear regression equations. The results were in good agreement with those reported by the reference method [11].

**Table 12:** Assay results for the determination of VPS in its co-formulated tablet with SFV by Method 1

Studied drug	Proposed method						Comparison method [11]
	Amount taken (ng/mL)		Amount found (ng/mL)		% Found		% Found
	Column (A)	Column (B)	Column (A)	Column (B)	Column (A)	Column (B)	
Epclusa^®^ tablet (SFV 400 mg/VPS100 mg)	1.0	2.5	0.982	2.449	98.20	97.99	102.45
	3.0	5.0	2.995	4.827	99.84	96.53	101.78
	5.0	10.0	5.049	9.782	100.97	97.82	98.91
Mean					99.67	97.45	101.05
±SD					1.39	0.80	1.88
t-test					2.74	3.05	(2.78)*
F-test					1.82	5.55	(19.0)*

Each result is the average of three separate determinations

* The figures between parentheses are the tabulated t and F values at P = 0.05 [17]

**Table 13:** Assay results for the determination of VPS and SFV in their co-formulated tablet by Method 2

Studied drug	Proposed method						Comparison method [11]
	Amount taken (ng/mL)		Amount found (ng/mL)		% Found		% Found
	Column (A)	Column (B)	Column (A)	Column (B)	Column (A)	Column (B)	
SFV Epclusa^®^ tablet (SFV 400 mg/VPS 100 mg)	200.0	200.0	196.900	199.520	98.45	99.76	99.98
	400.0	400.0	389.760	394.920	97.44	98.73	98.93
	600.0	600.0	596.760	589.080	99.46	98.18	100.83
Mean					98.45	98.89	100.85
±SD					1.01	0.80	0.95
t-test					1.83	1.42	(2.78)*
F-test					1.12	1.41	(19)*
VPS	50.0	50.0	48.810	49.955	97.62	99.91	102.45
Epclusa^®^ tablet (SFV 400 mg/VPS 100 mg)	100.0	100.0	98.000	98.040	98.00	98.04	101.78
	150.0	150.0	148.665	148.185	99.11	98.79	98.91
Mean					98.24	98.91	101.05
±SD					0.77	0.94	1.88
t-test					2.39	1.76	(2.78)*
F-test					5.89	3.99	(19.0)*

Each result is the average of three separate determinations

* The figures between parentheses are the tabulated t and F values at P = 0.05 [17]

#### Application of the proposed method for quality control of the studied drugs in commercial dosage forms

**Fig. 5:**
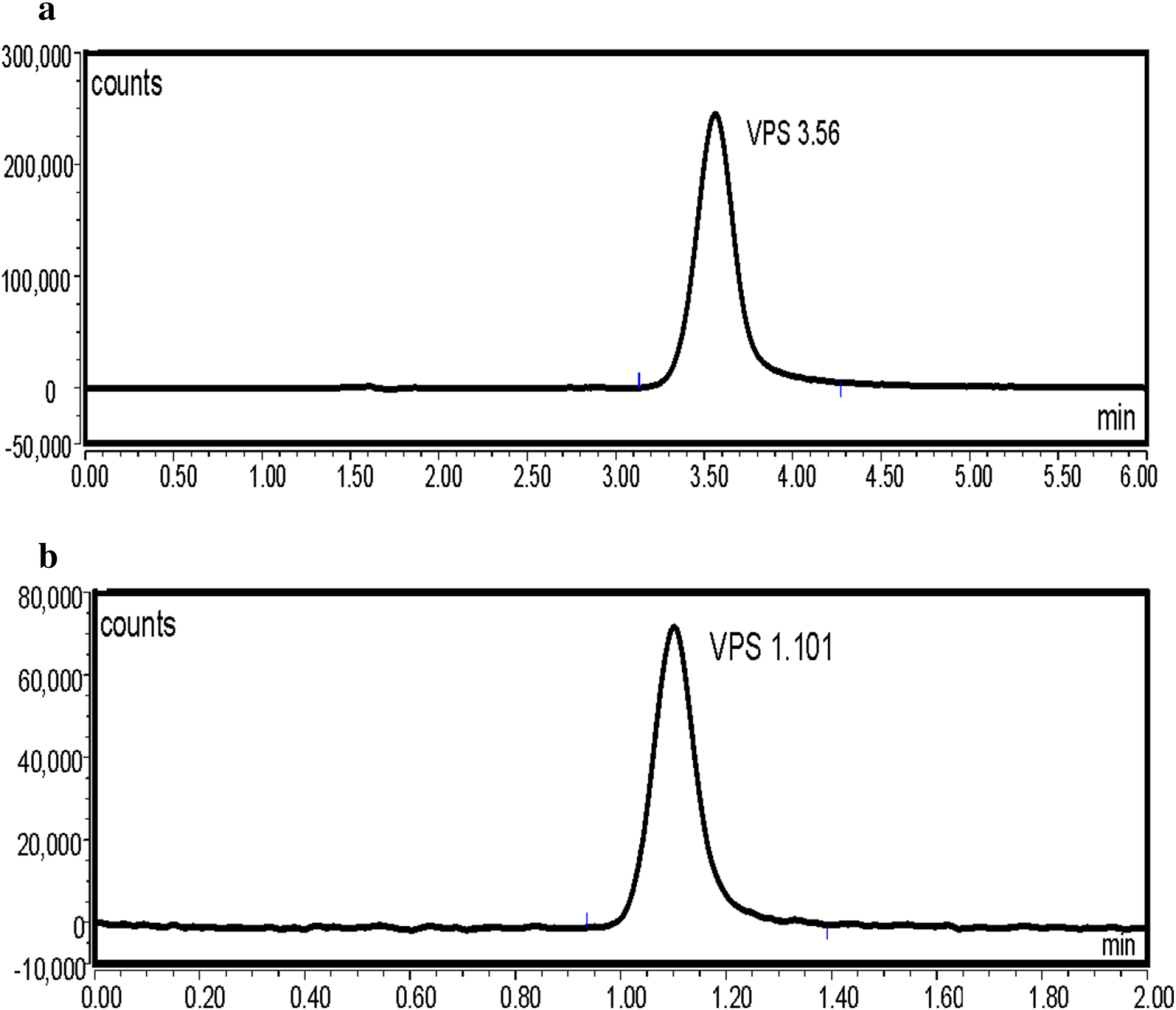
Typical chromatogram of VPS in its co-formulated tablet with SFV under the described chromatographic conditions (Method 1 A and B). **a** VPS 2.5 ng/mL utilizing column A. **b** VPS 1.0 ng/mL utilizing column B

**Fig. 6:**
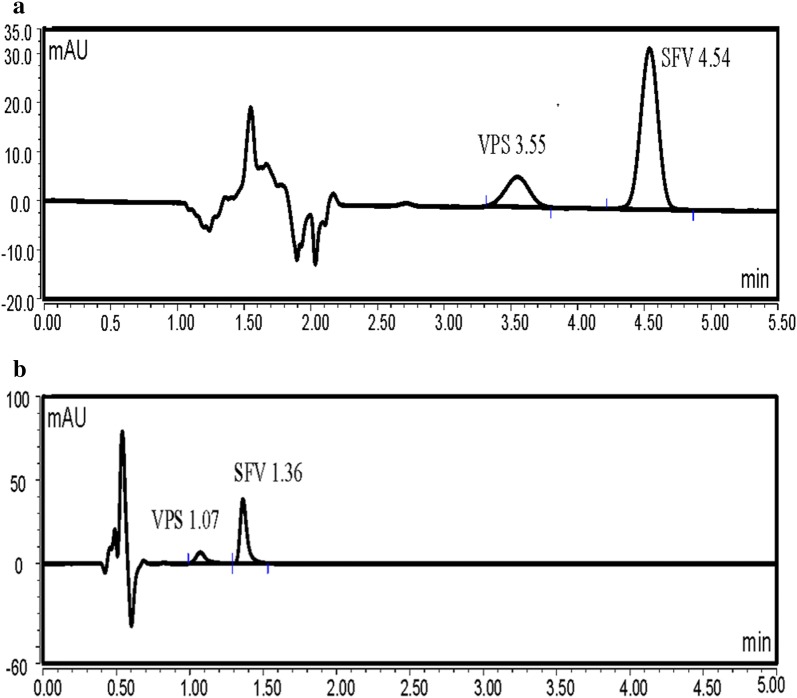
Typical chromatograms of co-formulated tablet of the studied drugs VPS (10 ng/mL) and SFV (40 ng/mL) under the described chromatographic conditions (Method 2A and B). **a** Method 2 utilizing column A. **b** Method 2 utilizing column B

The proposed methods were successfully applied for the determination of VPS and SFV in their commercially available co-formulated tablets (Figs. 5, 6). The results depicted in Tables 12 and 13 are consistent with those obtained using the comparison HPLC method [11]. Statistical analysis using Student’s t-test and variance ratio F-test [17] revealed no meaningful variation between the performance of the methods concerning the accuracy and precision, respectively. The favorable percentage recoveries with low standard deviation values emphasized that the proposed methods were convenient for the routine determination of the studied compounds in their commercial dosage form.

## Comparison of the two proposed methods

The present work describes two UPLC methods (1 and 2) with the utilization of two analytical columns (A and B) for the analysis of two antiviral drugs namely VPS and SFV.

Method 1 can detect only VPS without any interference from SFV, the method is highly sensitive when compared to Method 2 and far more selective. In addition, Method 1 A is more sensitive that Method 1 B, however Method 1 B provides shorter analysis time.

Method 2, has the advantage of being able to determine both drugs at the same time, the method is more sensitive when compared to previously reported ones, and provide short analysis time, where Method 2 B can resolve both drugs in less than 1.5 min. Both methods can be applied for quality control analysis of both drugs.

## Conclusion

The present work represented two convenient UPLC methods for the determination of VPS and SFV. The proposed UPLC approaches have been fully validated and demonstrated accurate assay methods for the determination of VPS and SFV with enhanced sensitivity and specificity. The good validation criteria of the proposed methods allow their application in quality control laboratories.

## Data Availability

All data and materials are available on request (Moustapha Eid Moustapha and Rania Mohamed El-Gamal).

## Abbreviations

VPS: velpatasvir
SFV: sofosbuvir
HCV: Hepatitis C Virus
US: United States
DAAs: Direct Acting Antivirals
AASLD: American Association for the Study of Liver Diseases
IDSA: Infectious Diseases Society of America
LC–MS/MS: liquid chromatography–tandem mass spectrometry
UPLC‐MS/MS: Ultra performance liquid chromatography-tandem mass spectrometry
RP-HPLC: reversed phase-high performance liquid chromatography
RSLC: rapid separation liquid chromatography
μL: micro liter
μg/mL: microgram per milliliter
v/v: volume per volume
mL/min: milliliter per minute
LOQ: limits of quantitation
LOD: limits of detection
ICH: The International Council for Harmonisation of Technical Requirements for Pharmaceuticals for Human Use
N: no. of theoretical plates
k’: capacity factor
Tf: tailing factor
S_a_: standard deviation of the intercept
S_b_: standard deviation of the slope
SD: standard deviation
%RSD: percentage relative standard deviation
%Error: percentage relative error
